# Supplementary material for: Are carbon emissions trading and green financial instruments synergistic? -Comprehensive quantitative research based on content analysis
Source: PLoS One. 2024 Mar 7;19(3):e0298601. doi: 10.1371/journal.pone.0298601 (PMC10919601; doi:10.1371/journal.pone.0298601)
Supplement: S1 Data — (ZIP) [file pone.0298601.s001.zip › Data/地级市Z_F工作报告.zip!/╕╩╦α/└╝╓▌╩╨/└┤╫╘╣1⁄2╓┌║┼í╛┬φ┐╦╩2╛▌═°í┐_2013.docx]

各位代表：

现在，我代表市人民政府向大会作工作报告，请予审议，并请各位政协委员和其他列席人员提出意见。

一、2012年工作回顾

2012年，在省委、省政府和市委的正确领导下，全市上下认真学习贯彻党的十八大精神，积极落实省上对兰州工作的一系列新要求，奋发向上，攻坚克难，经济社会发展保持增速较快、结构优化、民生改善、和谐稳定、转型跨越的良好态势，完成了市人大十五届一次会议确定的各项目标任务。

一年来，我们积极应对复杂的国内外经济形势，紧密结合兰州实际保增长、调结构，全市经济实现了平稳较快增长。针对宏观经济形势带来的影响，认真研究出台促进实体经济发展、提高经济首位度、加快“五个标准”建设、落实“3341”项目工程、扩大投资消费等政策措施，调高“十二五”规划相关指标，细化发展的阶段性目标和主攻方向，有效促进了经济社会较快发展。预计全市实现生产总值1570亿元，增长13.6%；工业增加值562亿元，增长12%；固定资产投资1220亿元，增长40%以上；社会消费品零售总额754亿元，增长18%；大口径财政收入406亿元，增长16%；公共财政预算收入103.73亿元，增长19.93%。多元支柱产业发展初见成效，凝炼储备产业项目1000多项，传统产业加快升级，新兴产业快速发展。特别是文化产业增加值达到30.9亿元，增长27%；旅游业迅猛发展，接待人数突破2000万人次，实现旅游收入155亿元，分别增长49.6%和50.6%。非公有制经济增加值占生产总值的比重提高到38.85%，单位生产总值能耗、污染减排等约束性指标全面完成，居民消费价格指数控制在3%以内。

一年来，我们深入开展“园区建设年”活动，全方位推动兰州新区、两个国家级开发区和八个县区产业园区建设，全市发展格局发生新变化。调整和加强了新区组织机构和领导力量，制定了新区总体规划和多个专项规划，基础设施建设和招商引资同步推进，“四纵四横”主干道、兰秦快速通道水秦段基本建成，舟曲灾后重建安置区、综合服务中心等投入使用，各项配套设施和社会服务功能加快完善，引进世界和国内民营500强企业16家，完成固定资产投资250亿元，实现生产总值116亿元。市区工业企业向新区积极拓展，兰电、农垦啤酒、际华三五一二、榕通管业搬迁改造项目开工建设，兰石、兰驼等企业出城入园工作积极推进。兰州新区已经成为我市转型跨越发展的主战场，呈现出明显的集聚效应和强劲的发展势头。高新区实现生产总值245亿元，增长28.9%。经济区实现生产总值102.2亿元，增长24.03%。各县区积极打造县区域核心产业园区，引进了一批符合园区规划和产业导向的项目，初步形成错位互补、多极突破、竞相发展的格局。

一年来,我们抢抓国家和省上支持兰州发展的政策机遇，积极开展“政策落实年”活动，取得了争取政策和项目的重大突破。在国家和省上的大力支持下，兰州新区成为全国第五个、西北第一个国家级新区，城市轨道交通项目正式开工建设，这是兰州和甘肃发展史上的两件大事。争取到全国大气污染联防联控重点城市、国家级低丘缓坡沟壑等未利用地开发试验区、国家级生物产业基地、全国“三网”融合试点城市等15个政策平台，国家和省上各类转移支付和专项资金92亿元，增长15.81%。所有这些，都为当前和今后的发展创造了千载难逢的机遇和条件。

一年来，我们紧盯大企业、大品牌、大项目，积极开展产业招商、以商招商和定点招商，招商引资成效显著，城市影响力持续扩大。工作中更加注重开放开发，积极发挥多重资源优势、综合成本优势和园区平台优势，先后组团赴外考察和举办项目推介活动20多次，对接国内外知名大企业70多家，吸引省外大型投资考察100多次，引进实施了铁建重工、中国四联、名城企业集团、碧桂园等一批大项目。全年引资2173.37亿元，到位资金790亿元，增长92.17%。坚持把办好节会赛事作为扩大开放的重要抓手，成功举办了第二届兰州国际马拉松赛，被中国田径协会评为年度“金牌赛事”。广泛开展“中国西北游、出发在兰州”宣传推介，有效扩大了城市知名度和影响力。

一年来，我们坚持以规划为引领，在治污染和畅交通上狠下功夫，城市形象和面貌得到改善提升。基本完成第四版城市总体规划，确定了新一轮城市发展的功能定位、规划范围和空间布局。全面开展大气污染防治整体战攻坚战，制定实施污染治理总体规划和5个专项方案，完成燃煤锅炉改造363台3064蒸吨，特别是进入供暖期后，采取24小时驻厂监察、电厂优质煤补贴、面源污染网格化管理、超标车辆限行等措施，城区空气质量明显改善，全年优良天数达到270天，比上年增加28天，优良率达到73.97%。制定实施“一横三环九纵”城市骨干路网规划，下大力气解决城区交通拥堵问题。南山路东段建成通车，中段和西段加快建设。北环路一期工程积极推进，二期工程开工建设。金雁黄河大桥建成通车，元通黄河大桥基本建成，深安黄河大桥加快建设。小西湖等5个立体交通节点改造启动实施。安宁至西站快速公交投入运营。城区停车场加快建设，新增停车泊位2156个。智能交通管理非现场执法系统一期建成运行。启动城市六大出入口改造工程，整治小街巷50条，建成城中村村民安置房100万平方米，新开工100万平方米。稳步推进城区大型批发市场外迁工作，完成大西北板材市场搬迁改造。

一年来，我们积极落实强农惠农富农政策，加大对“三农”的投入，农村生产生活条件有了新的改善。全市实现农业增加值45亿元，增长6%。高原夏菜、玫瑰、百合等特色农产品种植规模进一步扩大，新增设施农业7000多亩。推广全膜双垄沟播种植50万亩。三电、西电大型泵站完成年度改造任务，实施农村饮水安全工程14项，解决12万人饮水安全问题。建设养护农村公路1200公里。新建农村安居住宅14533户，完成农村危旧房改造7070户。新建改造农村便利超市100个，建成10个食品药品安全示范乡镇、100家食品药品放心消费示范企业和门店。编制实施区域发展与扶贫攻坚规划，启动七里河区黄峪乡整乡推进扶贫开发试点工作。城乡一体化试点工作实施27个重点项目，总投资30多亿元，通过了省上考核验收。积极推进“联村联户、为民富民”行动，2000个单位40000多名干部下基层结对开展联扶工作，全面落实“八个全覆盖”、“五件实事”等帮扶措施，兴办各类实事1600多件，帮助农民解决了一大批生产生活难题。

一年来，我们加强就业和社会保障工作，积极推进社会管理创新，城乡群众生活水平进一步提高。城乡居民收入分别增长15.5%和18.5％，是多年来增幅最高的一年。加大民生领域投入，为民兴办的十个方面28件实事全面完成。新增就业8.3万人，输转城乡富余劳动力34万人次，安置困难群众就业6000人。新开工建设保障性住房12874套，分配入住10288套。城市低保提高到五区每人每月354元、三县266元，农村低保每人每年1488元，全年为21.4万人发放救助保障金4.8亿元。全面落实低收入家庭冬季取暖补贴政策，为22.8万贫困居民发放取暖补贴4297.85万元。实行近郊四区70岁以上老年人免费乘坐公交车优惠政策。征收价格调节基金7530万元，支持了蔬菜直销店建设、平价肉菜进社区和冬春蔬菜储备。兰州新区舟曲中学建成启用，全市中小学D级危房全面消除，新建城乡幼儿园30所、乡村学校少年宫40个，启动校车专线130条，发放农村学生营养补贴6134万元。新农合扩面提标，补助标准人均提高到250元，参合农民受益率达100%。落实计划生育优惠奖励扶助政策，为流动人口提供了均等化服务。组建了兰州演艺集团，推出了《鼓舞中国》等精品剧目，支持了《黄河第一桥》剧目创作，兰州创意文化产业园被评为国家文化产业示范基地。建成200个城乡全民健身场地。“三维数字社会管理系统”列为国家标准，“虚拟养老院”服务人数达到18万人以上。依法打击违法犯罪活动，着力排查和化解矛盾纠纷，加强和改进信访工作，畅通群众诉求表达渠道，维护了社会和谐稳定。落实民族宗教政策，保持了民族团结、宗教和顺，启动了七里河“两个共同”示范区建设。强化国有资产管理，完成11户企业资产重组，解决了一批改制重组遗留问题。安全生产和食品药品监管工作得到加强。荣获“双拥模范城”七连冠。启动新一轮全国文明城市创建工作，打造了“凡人善举”、“兰州好人墙”等文明实践引领品牌，涌现出以“最美夫妻”李成环、龚大锬为代表的一批优秀人物，兰州在全国127个城市文明程度指数测评中，位列省会和副省级提名资格城市第七名。

一年来，我们认真实施“效能风暴”行动，大力改进工作作风，政府机关行政效能明显提升。主动接受人大法律监督和政协及社会各界的民主监督，办结人大代表意见建议186件、政协委员提案495件。在“效能风暴”行动中大力推进五项重点改革，新取消行政审批事项171项，成立了公共资源交易中心，政务服务中心按照“两集中、两到位”模式正式运行，实现跨部门行政执法联网和多部门协同审批，并将行政审批、重大项目、公共资源交易、行政处罚、行政投诉纳入电子监察。加大行政考核问责力度，对125名干部予以效能问责。深化政务公开，推进经济责任审计，政府系统廉政建设深入开展。统计、司法、人防、外事、气象、防震减灾等工作扎实推进，妇女儿童、老龄、残疾人、慈善事业等取得新进展。

各位代表，过去的一年，是我们在拼搏中前进、在转型中发展的一年，也是战胜各种挑战、实现重大突破的一年。各项工作取得的成绩，是省委、省政府和市委正确领导的结果，是市人大、市政协监督支持的结果，是全市各族人民团结奋斗、创业实干的结果。在这里，我代表市人民政府，向全市各族人民，各位人大代表和政协委员，各民主党派、工商联、人民团体和无党派人士，向离退休老同志和社会各界人士，向驻兰解放军指战员、武警官兵和公安干警，向所有关心支持兰州发展的朋友们表示衷心的感谢和崇高的敬意！

回顾一年来的工作，我们也清醒地认识到，当前发展中还面临不少矛盾和问题，主要表现在：省会城市的中心带动作用还发挥得不明显；全面小康实现程度存在短板，尤其是农民人均纯收入、文化产业增加值等指标欠账较大；经济发展受结构性矛盾的影响尚未根本转变，战略新兴产业和现代服务业支撑不足；城市功能和带动能力与在全省的龙头地位还不相适应，城市建设受资金的瓶颈制约较大，市场化融资的办法不多；破解难题任务依然艰巨，大气污染防治和畅交通仍需坚持打好整体战攻坚战；农村经济结构和农业种植结构调整步伐较慢，农村基础设施和公共服务不够完善，农民持续增收渠道不宽，扶贫攻坚任务繁重；一些部门和公务员思想解放不够、观念转变不快、工作能力不足、执行力不强，与转型跨越发展的现实需求还有差距。对此，我们将采取更加有力的措施，深入研究和加以解决，使政府工作更加符合发展的要求和人民的期盼。

二、2013年工作总体要求和发展目标

今年是全面贯彻党的十八大精神的开局之年，是深入落实省市党代会部署的关键之年，也是实施“十二五”规划承上启下的重要一年。

政府工作的总体要求是: 以党的十八大精神为指导，全面贯彻落实中央、省上和市委的决策部署，立足提前建成全面小康社会、提升经济首位度、提高西部省会城市排名，围绕“经济实力雄厚、生态环境良好、文明程度一流、建设水准较高、城市管理有序”的要求，深入实施“3341”项目工程，做快新区，做大产业，做美城市，做实民生，确保经济社会平稳较快健康发展，不断开创转型跨越、率先崛起的新局面。

经济社会发展的预期目标是：在更加注重发展质量和效益的基础上，确保生产总值增长15%；规模以上工业增加值增长14%；固定资产投资增长40%；社会消费品零售总额增长18%；大口径财政收入和公共财政预算收入分别增长18%，财政支出占全省比重达到12%；城镇居民人均可支配收入增长16%；农民人均纯收入增长18%；城镇登记失业率控制在4%以内；招商引资到位资金增长30%以上；居民消费价格涨幅控制在3.5%左右；节能减排完成国家和省上下达的目标任务。

工作的总抓手是：以实施“3341”项目工程为龙头，围绕招商引资实际利用资金突破1000亿元的目标，加快形成项目支撑发展的新格局。认真谋划和实施三大战略平台，经济平台重点发挥已有的近40个政策载体作用，抓好兰州新区建设和循环经济示范区建设，打造富有活力的增长极；文化平台按照创建华夏文明传承创新区的部署，充分发挥黄河文化为代表的资源优势，加快兰州都市圈文化产业区建设；生态平台围绕创建国家生态屏障建设保护与补偿试验区的目标，积极开展申报黄河中上游生态修复兰州试验区等工作。不断夯实三大基础建设，交通提升工程重点加快铁路枢纽、重要铁路、机场扩建和公路干线建设，构建立体化、网络化的大交通体系；信息畅通工程重点加快“数字兰州”建设，以信息化更好地支撑经济发展、社会管理和城市服务；城镇化建设重点走新型城镇化道路，加快形成老城和新区两轮驱动、县城和中心镇功能强化、农村环境大力改善的城镇化体系。紧紧瞄准战略新兴产业、特色优势产业、富民多元产业、区域首位产业，着力推进绿色发展、循环发展、低碳发展，不断发展壮大多元支柱产业。

三、举全市之力推进兰州新区建设

加快兰州发展，新区是关键。按照国家定位和规划方向，坚持以创新精神、借全省之力、奏开放强音、促产业集聚，高标准建设、新模式开发、大项目支撑，形成大城市、大产业、大要素的发展格局，打造兰州和全省发展的增长极。

加快产业向新区集聚发展。围绕石油化工、装备制造、高新技术、现代物流、现代农业和职业教育六大片区建设，大力推进产城融合，全面加快产业发展。力争铁建重工、大成聚光太阳能、华腾富世、亚太科技、吉利汽车扩能改造等项目建成投产。开工建设中国四联高新技术西北产业基地、南翔万商商贸物流集散基地等签约项目。对接抓好中国化工、柳工机械等在谈项目。强力实施市区工业企业出城入园，推进航天五一○所与真空设备厂整合重组新建项目，确保兰电、兰石等企业搬迁改造取得实质性进展。

推动项目向新区集中布局。以新区招商统领全市招商引资工作，在积极争取国家项目布局的同时，大力度大范围开展招商引资。围绕五大产业片区和七大主导产业，精心编制招商引资导则，明确产业链招商重点项目，严把项目准入关、环境评价关和投资强度关。瞄准全国各行业领军企业，采取点对点招商、节会招商、商会招商、龙头企业带动招商等模式，有计划、有选择、有目的地开展精确招商和高效招商，着力引进一批能拉长产业链条、提升产业层次、形成产业集群的爆发性增长项目。

引导城市功能向新区拓展完善。加快新区对外交通建设，兰秦快速通道水秦段建成通车，开工建设新区至白银公路联络线，建成马家坪至中川铁路，加快建设兰州至中川城际铁路。基本完成核心区基础设施建设任务，完善产业园区综合路网框架，加大市政公用配套设施建设力度。完成2个生态湖、湿地公园、市民广场等重点项目。积极推进学校、医院等社会服务设施建设，建成兰州实验幼儿园新区分园等项目。加强农民就业技能培训，引导向非农产业转移，妥善解决好被征地农民就业安置和养老保险问题，建成农民安置房100万平方米以上。

促进工作重心向新区倾斜转移。按照新调整的园区管理体制，将高新区和经济区开发建设重点向新区倾斜，推动“三区”融合发展。深入挖掘国家和省上支持政策内涵，积极探索多种渠道的市场化融资模式，打造功能强大的投融资平台，广泛吸引金融资金、社会资本参与新区建设。采用“飞地经济”等开发模式，吸引省内兄弟市州参与新区开发。强化“特区”意识，发挥政务服务中心功能，推行一站式、直通车高效服务，开辟项目引进绿色通道，打造政策洼地和服务高地。

四、在产业结构调整中加快转变发展方式

立足产业基础和发展优势，把多元支柱产业融入“3341”项目工程四大产业方向，培育战略新兴产业，发展特色优势产业，壮大多元富民产业，提升区域首位产业，着力培育新的经济增长点。

培育战略新兴产业。充分发挥区域性科技资源优势，在生物医药、新材料、节能环保、信息技术、新能源、航空航天等六大领域，实施新兰药异地扩建、蓝星碳纤维二期、中德环保垃圾处理、信源软件、振发光伏发电、中航电机等54个重点项目。抓住政策机遇，突出打造国家级生物产业基地，壮大生物特色产业，发展生物服务业，形成生物医药、生物医学工程、生物育种等产业集群，努力实现规模化、集聚化和国际化发展。

发展特色优势产业。以连海开发区、金崖钢铁基地、金川科技园为重点，实施连铝、兰铝电解槽技术改造、金川电池材料创新示范园等重大项目，壮大有色冶金产业规模。加快建设北车高端轨道交通装备制造基地，促进形成实力较强的装备制造产业集群。依托循环经济示范基地建设，重点打造石油化工等7条循环经济产业链，推进实施窑街煤电公司煤电一体化项目。支持甘肃烟草公司新建高档卷烟制丝生产线。

壮大多元富民产业。坚持发挥农业资源优势，突出特色，找准定位，积极推进农业转型升级。做大做强特色种植业，力争高原夏菜、百合、玫瑰、中药材等种植面积突破150万亩，启动现代都市生态农业示范园区建设。培育发展规模养殖业，新建规模养殖场40个，新增畜禽养殖量100万头只以上，大幅提高畜牧业在农业中的比重。打造农副产品加工产业集群，扶持龙头企业、农民专业合作社发展，支持建设一批农副产品深加工生产线。发展农产品现代物流业，扩大特色农产品外销，提高在全国市场的影响力。

提升区域首位产业。发挥中心城市要素集中的优势，积极引进战略投资者和知名品牌，大力发展商贸、金融保险、会展商务、公用服务等先进业态，培育一批功能集中、配套完善的特色商圈。坚持规划引领、政府主导、企业主体、市场运作，编制完成全市物流产业发展总体规划，加快实施一批物流重点项目，推进大型批发市场外迁工作。依托在兰央企综合优势，加快实施1000万吨炼油、1000万吨原油储备库、碳五加氢、丁基橡胶等重点项目，全力推进石油化工产业链延伸，建设国家石油化工基地、战略性石油储备基地。

五、建设宜居宜业宜游美丽兰州

坚持生态文明的发展理念，突出以人为本的建设导向，着力破解大气污染、交通拥堵等“城市病”，不断提升城市形象品位，增强城市的集聚力、辐射力和带动力。

继续打好大气污染防治整体战攻坚战。坚持“三步并作两步走”，实施370台27665蒸吨生活锅炉和31台203蒸吨工业锅炉改造，提前一年完成全部改造任务。加快实施大唐热电等12个工业治污项目，淘汰新陇铁合金等一批落后产能。全面整治车辆超标排放问题，建立低标号燃油退市和“黄标车”淘汰机制，试点推广新能源汽车。加强扬尘整治和管理，有效消减各类扬尘污染。提高环境监测能力，强化对重点污染企业的动态监管。抓好治污立法工作，制定出台燃煤管理条例、工业企业排放标准等地方性法规。力争城区空气质量优良天数达到292天、优良率达到80%，进入全国空气质量达标城市行列。

加快公共交通基础设施建设。以城市轨道交通建设为龙头，统筹安排路网建设和畅交通工作。精心做好轨道交通开工准备，全线开工建设1号线一期工程。构建“一横三环九纵”骨干路网体系，力争南山路、北环路建成通车，形成城市二环路。配合实施南绕城、北绕城高速工程，加快构建城市三环路。打通盐场路、火车站南路、榆中街等16条断头破损道路。优化背街小巷交通微循环功能。加快推进小西湖等5个立体交通节点改造。大力发展公共交通，着力优化公交线网站点布局。建立公共自行车租赁系统，鼓励和引导绿色出行。积极支持兰渝铁路、兰新第二双线、宝兰客运专线、西客站、中川机场扩建等项目建设。

推进老城区改造提升。拓展老城区发展空间，全力加快东部科技新城建设，打造城市空间拓展区、创新要素聚集区、高新技术示范区。全面推进国家级低丘缓坡沟壑等未利用地综合开发试验区建设。继续实施城中村改造，建成村民安置房100万平方米、新开工100万平方米。建设城市规划展览馆、科技馆、马拉松公园、体育中心等公共设施项目。加快万达广场、鸿运金茂、红楼时代、国际商贸中心等高端城市综合体和地标性项目建设。全面实施城市出入口综合整治，打造富有特色的城市窗口形象。

加强生态环境建设。制定实施生态战略平台行动计划，优化全市林业生态建设布局，启动百万亩林业生态屏障、百万亩林果产业基地、百万亩森林资源保护三大工程。全面推进黄河风情线改造提升，实施徐家湾旅游文化风情区等重点项目，新建一批小游园和绿色景观。加强南北两山生态景观建设，构建市区至新区绿色长廊。全年新增造林绿化面积10万亩，其中兰州新区5万亩，经济林占到一半以上。加快中铺子生活垃圾发电项目建设，提高垃圾无害化处理和资源化利用水平。

推进城市精细化管理。增强规划的前瞻性和约束性，争取批准实施第四版城市总体规划，编制完成市域城镇体系规划，加强山水城市、环兰州城市群等重点规划研究。加快“数字兰州”建设，整合城市管理视频资源、专线资源和工作力量，建成全市统一的三维数字社会服务管理平台。推行城市扁平化管理，充分发挥街道社区在城市管理中的重要作用。深入推进智能交通建设，优化交通管理，缓解交通拥堵。集中力量整治市民反映突出的物业管理、供热服务等问题，整治改造165个老旧住宅小区。

六、推进城乡发展一体化

围绕提前建成全面小康社会，坚持现代农业的发展方向，注重把工作精力向“三农”倾斜，加大支持力度，夯实发展基础，增强农村发展活力。
